# Predictors of Peripheral Neuropathy in Metabolic Disease: A Multivariable Analysis Incorporating the Toronto Clinical Scoring System and Sudomotor Assessment

**DOI:** 10.3390/medicina62030586

**Published:** 2026-03-20

**Authors:** Cristina Mocanu (Chitan), Radu-Cristian Cimpeanu, Teodor Salmen, Marius-Costin Chitu, Raluca-Elena Alexa, Claudiu Cobuz, Vasilica Cristescu, Anca Pantea Stoian, Cristian Serafinceanu

**Affiliations:** 1 Doctoral School, Carol Davila University of Medicine and Pharmacy, 020021 Bucharest, Romania; cristina.mocanu-chitan@drd.umfcd.ro (C.M.);; 2 Outpatient Diabetes Center 1, Suceava County Emergency Clinical Hospital “Sf. Ioan cel Nou”, 720224 Suceava, Romania; 3 Department of Emergency Medicine, Clinical Emergency County Hospital, 200642 Craiova, Romania; 4 Department of Infectious Disease, “Dr. Carol Davila” Central Military Emergency University Hospital, 010825 Bucharest, Romania; 5 Cantacuzino National Military Medical Institute for Research and Development, 050096 Bucharest, Romania; 6 Grigore T. Popa University of Medicine and Pharmacy, 700115 Iași, Romania; 7 Faculty of Medicine and Biological Sciences, Ştefan cel Mare University of Suceava, 720229 Suceava, Romania; claudiu.cobuz@usm.ro; 8 School of Medicine and Pharmacy, University Titu Maiorescu, 040441 Bucharest, Romania; 9 Department of Diabetes, Nutrition and Metabolic Diseases, “Carol Davila” University of Medicine and Pharmacy, 020021 Bucharest, Romania; anca.stoian@umfcd.ro (A.P.S.); cristian.serafinceanu@umfcd.ro (C.S.)

**Keywords:** peripheral neuropathy, diabetes mellitus, TCSS, Sudoscan

## Abstract

*Background and Objectives:* Peripheral neuropathy (PNP) is a frequent and debilitating complication among patients with diabetes mellitus (DM) and other metabolic conditions, substantially affecting morbidity, functional status, and quality of life. Identifying predictors of PNP is essential for optimizing early diagnostic strategies and improving long-term management outcomes. The aim of this study was to determine the predictive factors of PNP in a cohort of patients with DM. *Materials and Methods:* A cross-sectional study including 117 patients diagnosed with DM assessed for PNP was conducted. All patients were evaluated clinically and biologically. PNP was clinically assessed using the Toronto Clinical Scoring System (TCSS) score and sudomotor function by Sudoscan. *Results:* The patients included were mostly males with type 2 DM and metabolic syndrome phenotypes. Moreover, the patients with PNP were much older than those without PNP (65 [57–69] vs. 59.50 [46–68] years, *p* = 0.008), with a longer duration of DM (10 [6–15.50] vs. 5.5 (2–14] years, *p* = 0.019), and associated autonomic diabetic neuropathy (χ^2^ = 24.382, *p* < 0.001). Furthermore, TCSS and Sudoscan were correlated with a history of PNP, especially Sudoscan, which showed a very good discriminative ability for diabetic neuropathy diagnosis (AUC = 0.816). In a multivariable logistic regression including age, DM duration, and HbA1c, age was independently associated with PNP, with each additional year increasing the odds of neuropathy by approximately 6% (OR = 1.06, 95% CI 0.02–1.09, *p* = 0.002). When age was excluded, DM duration showed a borderline association with PNP (OR = 1.055, CI95% 0.997–1.117), suggesting potential overlap between these variables. Adding sudomotor assessment to the initial model improved the model performance (AUC 0.70–0.72). *Conclusions*: Age emerged as the main independent predictor of diabetic neuropathy, highlighting the role of cumulative metabolic exposure in the development of neural damage. Moreover, sudomotor assessment may have a complementary role in PNP assessment.

## 1. Introduction

Peripheral neuropathy (PNP) represents one of the most common and disabling complications affecting patients diagnosed with chronic metabolic diseases, particularly diabetes mellitus (DM) [1]. Its presence significantly elevates the risk of severe secondary conditions, including foot ulceration, infections, and lower-limb amputation, which define the clinical syndrome known as diabetic foot [1,2].

According to the World Health Organization and the International Working Group on the Diabetic Foot, the diabetic foot is characterized as a complex neuro-osteo-arthropathic condition, with or without ulceration, involving pathological alterations in the skin, soft tissues, musculature, joints, and bone structures [2,3,4]. This syndrome is primarily driven by the presence and severity of PNP, often in conjunction with peripheral artery disease, and represents a high clinical and economic burden due to its invalidating complications such as neuropathic pain, tissue infections, ulceration, and amputations that lead to decreased quality of life and, subsequently, to absences and amputations [5,6,7,8,9].

The prevalence of diabetic PNP is similar in type 1 and type 2 DM, and varies widely from 7 to 90% due to limited studies in some regions of the globe [10]. This heterogeneous variation in prevalence is secondary to multiple factors, such as age, present comorbidities, and duration of DM evolution, but also due to unstandardized diagnostic methods and criteria [8,11,12,13]. Despite regional variations, its prevalence is consistently described as one of the most frequent neurological disorders encountered in clinical practice, surpassed only by cerebrovascular disease and herpes zoster [2,6,14]. Despite its high frequency, approximately 30% of neuropathy cases remain idiopathic, demonstrating the incomplete understanding of its underlying etiology [15]. Growing evidence suggests that advanced age, chronic hyperglycemia, and metabolic–inflammatory mechanisms play central roles in nerve injury and degeneration [2,6]. Also, in terms of treatment, the available tools are scarce, and a clear management plan is hard to establish [8,9,10,11,12,13].

In recent years, a considerable body of evidence has highlighted the contribution of visceral adiposity to the pathogenesis of neuropathy [16]. Excess abdominal fat has been shown to promote chronic low-grade inflammation, dysregulated adipokine secretion, insulin resistance, and oxidative stress, all of which contribute to peripheral nerve dysfunction [4,6,17]. These mechanisms are increasingly recognized as potential drivers of neuropathy even among individuals without DM. This suggests that age and glucose control are not the only major determinant elements by themselves, but the duration of exposure to metabolic factors that are not strictly controlled has an even more significant role in the development of severe complications of neuropathy [18,19,20].

This study aims to investigate clinical and biological predictors of PNP in patients with DM, with a particular focus on metabolic markers such as HbA1c, body mass index (BMI), and waist circumference (WC), as well as the use of the Toronto Clinical Scoring System (TCSS) and Sudoscan analysis in evaluating and grading diabetic neuropathy severity. Because clarifying their associations may improve early detection and refine clinical management strategies for preventing neuropathy-related complications, we hypothesized that the addition of a sudomotor assessment (Sudoscan) to classical clinical parameters would significantly increase the predictive accuracy for PNP diagnosis.

## 2. Materials and Methods

This cross-sectional observational study was carried out according to the STROBE (Strengthening the Reporting of Observational Studies in Epidemiology) guidelines, which were the framework on which this section was developed [21].

### 2.1. Study Population

Consecutive patients presented to two diabetologists in the Outpatient Department of Diabetes at Emergency County Clinical Hospital St. Ioan cel Nou, Suceava, Romania, between 1 September and 30 September 2025, with 5 outpatient days a week, were evaluated per day for inclusion in the study.

### 2.2. Inclusion and Exclusion Criteria

#### Inclusion Criteria

All participants were adults with previously established diagnoses of DM of at least 1 year, with a complete biologic profile, with positive diagnoses of PNP established by a neurologist, and providing signed informed consent.

Patients were excluded if they did not have a PNP diagnosis from a neurologist and if they had a secondary cause of neuropathy, such as a history of lumbar disc disease, autoimmune disorders, toxic exposures, or non-metabolic causes, such as alcohol consumption, vitamin B12 deficiency, infectious etiologies, or a history of cancer and radiotherapy or chemotherapy.

### 2.3. Clinical Assessment

A complete medical history was conducted, focusing on autoimmune disorders, toxic exposure, lumbar disc disorders, infectious disorders, and alcohol consumption, in order to identify other causes for distal neuropathy. Furthermore, a history of chronic coronary syndrome, chronic venous disease, trophic lesions, and ulcerations was attained.

Regarding medication, of interest were hypoglycemic medications. Also, the patients were asked whether they had taken medication for neuropathy previously in the last 3 months. In patients with cancer, a history of radiotherapy or chemotherapy was obtained.

Weight, height, and WC were measured while patients were barefoot and wearing light clothing. BMI was calculated as weight/height^2^ (kg/m^2^) and WC was measured at the midpoint between the inferior rib margin and superior iliac crests.

Blood pressure was measured after 10 min of rest, with the patient sitting in a back-supporting position, with their legs uncrossed, feet flat on the floor, and arm at heart level. There were 2 measurements obtained and the mean was used for the current study.

All patients were evaluated for peripheral artery disease through ankle–brachial index measurement. The patients sat in a supine position, and using a portable Doppler ultrasound and blood pressure cuffs, systolic blood pressure was measured in both arms and legs. The ankle–brachial index was obtained as the ratio between the ankle and brachial systolic blood pressure.

### 2.4. Biological Assessment

The biological assessment included HbA1c (normal values < 5.7%), total cholesterol (normal range: 50–200 mg/dL), LDL-cholesterol (normal value according to cardiovascular risk score, mostly <55 mg/dL), triglycerides (normal range: 50–150 mg/dL), vitamin B12 (normal range: 187–820 pg/mL), and urinary albumin–creatinine ratio (normal value < 30 mg/dL).

### 2.5. Polyneuropathy Assessment

A PNP-positive diagnosis was made previous to inclusion in the study by a neurologist. Furthermore, patients with a history of PNP were evaluated using the TCSS, which integrates symptom intensity, sensory testing, and reflexes to produce a standardized severity score. The patients were evaluated on the same day but not at a specific time of the day, in the same consulting room, at a constant temperature of around 20–22 °C by TCNS and then by Sudoscan. TCNS evaluated limb symptoms as 0—normal or 1—abnormal or weak; feel as 0—normal or 1—abnormal or weak; and reflex as 0—normal, 1—abnormal or weak, or 2—absent. TCNS scores range from 0 to 19: 0–5 indicates no DPN, 6–8 indicates mild DPN, 9–11 indicates moderate DPN, and 12–19 indicates severe DPN.

Furthermore, to better evaluate and grade the severity of PNP, a Sudoscan assessment was performed. Sudoscan evaluates neuropathy reflected by sudomotor dysfunction using electrochemical skin conductance (ESC), measured in microSiemens (µS), and is the ratio of generated current to a constant DC stimulus (≤4 V) applied to electrodes. The ESC values are categorized as >60 µS for no dysfunction, 60–40 µS for moderate dysfunction, and <40 µS for severe dysfunction [22].

### 2.6. Ethical Approval

This study was conducted according to the Helsinki Declaration and received ethical approval by the Ethical Committee of the Emergency County Clinical Hospital “Sfântul Ioan cel Nou” Suceava, number 36/28.08.2025.

### 2.7. Statistical Analysis

Statistical analysis was performed using IBM SPSS version 29.0 (IBM Corp., 2023, New York, NY, USA). Continuous variables were assessed for normality using the Shapiro–Wilk test and are presented as mean, standard deviation, or median and interquartile range. Categorical variables are expressed as frequencies and percentages. Comparisons between groups were conducted using the independent samples *t*-test or Mann–Whitney U test for continuous variables, and chi-square test for categorical variables. Correlations were evaluated using Spearman’s rank correlation coefficient.

Univariate comparison was considered exploratory and was used to identify factorpotentially associated with PNP prior to multivariable modeling. The main inferential analysis was based on the multivariable logistic regression model.

The discriminative ability of TCSS and sudomotor function (Sudoscan) was evaluated through receiver operating characteristic (ROC) curve analysis. Multivariable logistic regression was used to identify factors independently associated with PNP. Model discrimination was evaluated using the area under the ROC curve (AUC), while calibration was assessed with the Hosmer–Lemeshow test. Model adequacy was evaluated using Events Per Variable (EPV) criteria. Since the primary multivariable model included 71 neuropathy events and 3 predictors, the EPV was 23.7, exceeding the commonly recommended minimum number of 10. The extended model, including Sudoscan and EPV, remained adequate (17.8), supporting the stable estimation of odds ratios. Moreover, all predictors included in model 1 (age, DM duration, HbA1c) showed very low variance inflation factor values (age VIF = 1.143, DM duration VIF = 1.073, HbA1c VIF = 1.189). These results indicate no relevant multicollinearity between variables.

Regression coefficients, odds ratios, 95% confidence intervals, and *p*-values were reported. A *p*-value < 0.05 was considered statistically significant.

## 3. Results

**Figure 1:**
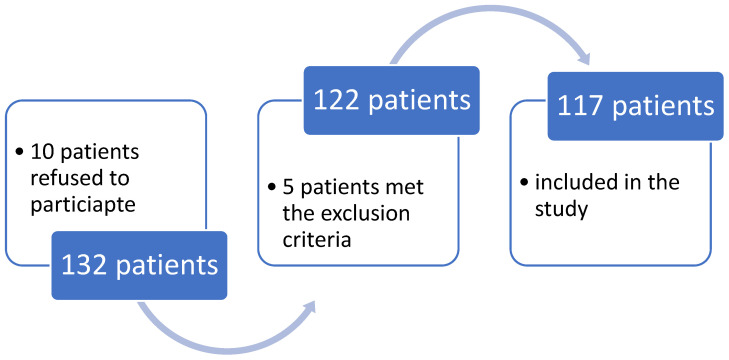
Flowchart of selection of the included patients.

From 132 eligible patients, 10 refused to participate, while 5 patients met the exclusion criteria, so only 117 patients were included, as shown in Figure 1.

### 3.1. Cohort’s Characteristics

The patients included were 59 years old (59.12 ± 1.24 years old), mostly males, with the metabolic syndrome phenotype (obesity, poor glycemic control, and altered lipid profile). Most of the patients had PNP (60.7%). Regarding other complications of DM, the patients presented with autonomic diabetic neuropathy (38.5%), microvascular complications (retinopathy—29.1%, nephropathy—9.4%, peripheral artery disease—17.9%), and stroke (19.7%). Table 1 summarizes the main characteristics of the studied group.

### 3.2. Correlations Between PNP and Clinical and Biological Parameters

Table 2 presents a comparison between patients with neuropathy and without neuropathy.

No significant differences were observed between gender (χ^2^ = 0.085, *p* = 0.770), urban settlement (χ^2^ = 2.634, *p* = 0.105), DM type (χ^2^ = 3.282, *p =* 0.070), presence of diabetic retinopathy (χ^2^ = 0.325, *p =* 0.569), diabetic nephropathy (χ^2^ = 2.273, *p* = 0.197), BMI (30.07 ± 0.65 vs. 30 ± 0.80, t = −0.058, *p =* 0.954), WC (101.38 ± 1.69 vs. 101.78 ± 2.32 cm, t = −0.212, *p =* 0.832), total cholesterol (177.20 ± 5.28 vs. 188.94 ± 7.46 mg/dL, t = 1.320, *p =* 0.190), LDL-cholesterol (110.53 ± 4.43 vs. 123.78 ± 6.98 mg/dL, t = 1.602, *p* = 0.113), and, respectively, HbA1c (t = −0.760, *p =* 0.449).

Patients with PNP were much older than those without PNP (65 [57–69] vs. 59.50 [46–68] years, *p =* 0.008), with a longer duration of DM (10 [6–15.50] vs. 5.5 (2–14] years, *p =* 0.019), and associated autonomic diabetic neuropathy (χ^2^ = 24.382, *p <* 0.001), as shown, also, in Table 2.

### 3.3. Correlations Between PNP, TCSS, and Sudoscan

The patients with a history of PNP had significantly higher TCSS scores than those without (8 [5,6] vs. 5 [6–9], *p* < 0.001). Moreover, TCSS showed a moderate negative correlation with the mean results from the Sudoscan examination of the feet (Spearman’s r = −0.442, *p* < 0.001), indicating that the neuropathy’s severity was associated with the worst sudomotor function.

**Figure 2:**
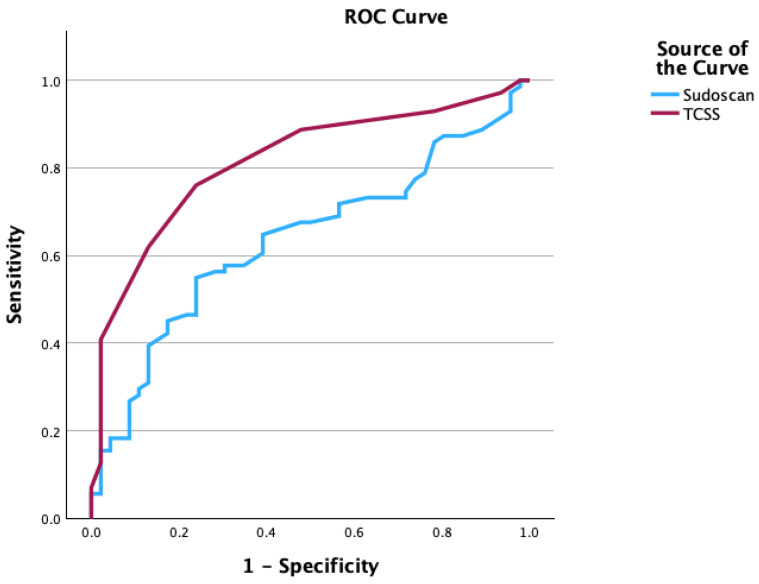
ROC curve for TCSS and Sudoscan in the diagnosis of peripheral neuropathy (AUC for TCSS = 0.816, AUC for Sudoscan = 0.637).

The feet ESC showed a modest discriminative ability for diabetic neuropathy (AUC = 0.637) compared with TCSS, which showed a very good discriminative ability for diabetic neuropathy’s diagnosis (AUC = 0.816), suggesting that Sudoscan can be used as a complementary test for PNP diagnosis, as shown in Figure 2.

Moreover, no significant correlation was found between the ankle–brachial index and neuropathy assessed by TCSS (r = −0.062, *p* = 0.508) or Sudoscan (r = 0.029, *p* = 0.753), suggesting that macrovascular disease may not directly influence neuropathic changes.

### 3.4. Predictors for PNP

In the multivariable logistic regression analysis including age, DM duration, and HbA1c, age was independently associated with PNP. Each additional year was associated with approximatively 6% higher odds of neuropathy (OR = 1.06, CI95% 1.02–1.09, *p* = 0.002). DM duration and HbA1c were not independently associated with neuropathy in the adjusted model. This clinical model demonstrated an acceptable-to-good discriminative ability (AUC = 0.70), with an adequate calibration (Hosmer–Lemeshow *p =* 0.070).

**Figure 3:**
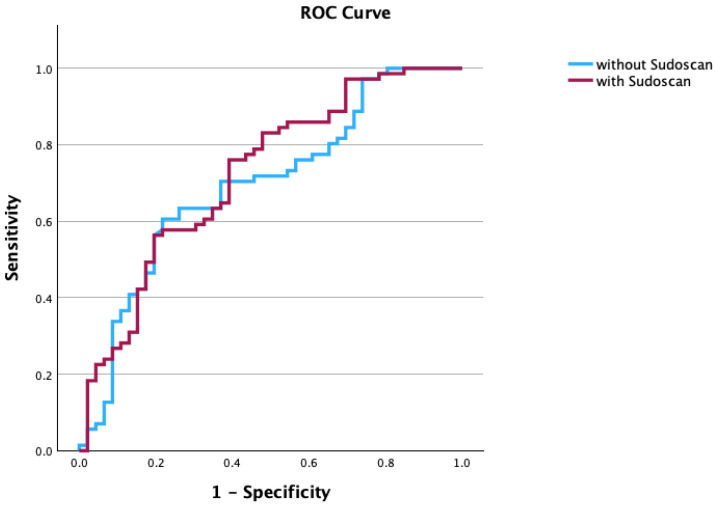
ROC curve for model 1 (including age, DM duration, and HbA1c) and model 2 (model 1 + Sudoscan results).

Although Sudoscan was not independently associated with neuropathy, its inclusion slightly increased the AUC from 0.70 to 0.72, as shown in Figure 3.

These findings suggest that functional sudomotor assessment may provide incremental value beyond traditional clinical variables.

When age was excluded from the initial model, DM duration showed a borderline association with PNP (OR = 1.055, CI95%: 0.997–1.117), suggesting potential overlap between these variables.

## 4. Discussion

The findings of this study are consistent with the established literature indicating that PNP, in metabolic diseases, arises from a complex interaction of metabolic, inflammatory, and neurodegenerative processes. The identification of age as a significant predictor, while HbA1c associated with higher odds of PNP, reinforces the well-documented roles of aging and chronic hyperglycemia in nerve degeneration. The association between TCSS scores and PNP confirms the clinical relevance of this standardized instrument for identifying both the presence and severity of PNP.

PNP is one of the most prevalent and disabling complications of DM and other metabolic disorders, with major implications for morbidity, quality of life, and healthcare burden [1,6,14].

PNP affects between 2% and 7% of the general population and occurs with markedly greater frequency in individuals diagnosed with DM. Epidemiological studies indicate that diabetic PNP affects between 8% and 54% of patients with type 1 DM and between 13% and 46% of those with type 2 DM, positioning it as the third most prevalent neurological disorder worldwide. Despite this high prevalence, its etiological factors remain undetermined in a substantial proportion of cases, reinforcing the multifactorial nature of PNP [6,14,23,24,25].

From a pathophysiological perspective, diabetic PNP results from a combination of biochemical and metabolic disturbances triggered by chronic hyperglycemia [6,26,27,28]. These include axonal degeneration, demyelination, activation of the polyol pathway with subsequent accumulation of sorbitol and fructose, mitochondrial dysfunction, oxidative stress, microvascular impairment, and chronic systemic inflammation [6,26,27,28,29,30]. Together, these processes lead to progressive sensory loss, reduced nerve conduction velocity, motor deficits, and autonomic dysfunction [6,14,28,29,30,31,32].

Clinical assessment of neuropathy relies on a combination of subjective symptom evaluation and objective testing [6,25]. Among the validated instruments, the TCSS integrates symptom assessment, sensory examination, and tendon reflex evaluation to quantify neuropathy severity [6,25,32,33,34,35]. Additional neurophysiological tests, such as nerve conduction studies, also contribute to diagnostic accuracy; however, these are more commonly employed in the advanced stages [6,36].

Increasing attention has been directed toward the role of visceral adiposity in PNP [37]. WC, an accessible proxy for visceral fat, has been associated with elevated levels of pro-inflammatory cytokines, metabolic dysregulation, and oxidative stress [38,39,40]. Notably, longitudinal studies have shown that even small increases in WC correlate with increased neuropathy risk, independently of glycemic control [41,42,43]. This association helps explain why strict glycemic management substantially reduces PNP risk in type 1 DM but yields more modest benefits in type 2 DM, where visceral adiposity and chronic inflammation exert greater influence [44,45,46].

Given the multifactorial etiology of PNP and the complex interactions among metabolic, anthropometric, and inflammatory processes, an integrated analytic approach is necessary to better understand the predictors of neuropathy in clinical populations [47].

The findings of this study are consistent with the established literature indicating that PNP, in metabolic diseases, arises from a complex interaction of metabolic, inflammatory, and neurodegenerative processes [29,48]. The identification of age and HbA1c as significant predictors reinforces the well-documented roles of aging and chronic hyperglycemia in nerve degeneration [49,50]. The association between TCSS scores and PNP confirms the clinical relevance of this standardized instrument for identifying both the presence and severity of PNP [51,52].

In our current cross-sectional study, age emerged as the only independent factor associated with PNP in multivariable logistic regression, whereas DM duration showed a borderline association after adjustment.

The strong predictive effect of neurotrophic treatment likely reflects clinical reality rather than causality. Neurotrophic agents are typically prescribed after neuropathy becomes symptomatic or clinically evident, meaning that this variable may serve as a surrogate indicator of neuropathy severity or symptom burden. Nonetheless, its statistical significance underscores the importance of accounting for treatment history when examining predictors of neuropathy.

The borderline association of BMI and WC with neuropathy is of particular interest. The inverse trend observed for WC, though not statistically significant, diverges from prior evidence linking visceral adiposity to increased neuropathy risk. Several explanations are plausible. First, confounding factors such as differential treatment, medication regimens, or comorbidities may have influenced our findings. Second, WC may not perfectly capture visceral fat distribution in all individuals. Third, the complex metabolic and inflammatory environment in obesity may generate heterogeneous neuropathy phenotypes that require further exploration.

The regression coefficients and confidence intervals support the conclusion that age is an independent predictor of PNP, while anthropometric measures exhibit marginal effects, and HbA1c, neuropathy severity, and neurotrophic treatment are associated with higher odds of neuropathy severity.

### 4.1. Advanced Diffusion Magnetic Resonance Imaging (MRI) and Peripheral Nerves 

A novel tool proposed as a sensitive biomarker of microstructural neuronal integrity is advanced diffusion MRI of peripheral nerves [53,54]. In a recent study by Pušnik et al. [55], high-field MRI that evaluated diffusion tensor imaging metrics of the sural nerve proved that it varied significantly between patients with type 2 DM versus patients without DM, showing lower fractional anisotropy and higher mean difussivity and radial difussivity. Also, they proved a correlation for histological markers of reduced fiber density and lower myelin proportion. This aspect emphasizes that diffusion metrics can capture axonal- and myelin-related alterations, modifications that may appear previously or be associated with conventional clinical staging. Even though these findings were obtained in ex vivo conditions and at an ultra-high field strength, high-field diffusion MRI techniques seem increasingly feasible in vivo, suggesting potential future applicability of diffusion tensor imaging-based biomarkers in the early and noninvasive assessment of PNP in type 2 DM [56,57].

### 4.2. Age as a Proxy for Cumulative Exposure

The multiple neuropathy-relevant insults beyond glycemia alone (long-standing dyslipidemia, high blood pressure, obesity-related inflammation, insulin resistance and microvascular dysfunction) took place in a duration-dependent manner throughout DM’s evolution [58,59]. This concept is consistent with the fact that distal symmetric PNP in DM is multifactorial and beyond HbA1c in cross-sectional samples, particularly in populations with heterogeneous cardiometabolic burden and treatment regimens [60,61]. Therefore, even if HbA1c proved to have a limited independent contribution in the adjusted model, it should not be interpreted as the absence of biological relevance, but rather as reflecting confounding by cumulative exposure, comorbidity clustering, and potential measurement timing relative to the chronic nature of nerve injury [58,59,60,61,62,63].

Current clinical guidelines, including the recommendations from Diagnosis and Treatment of Painful Diabetic Peripheral Neuropathy, emphasize the importance of systematized clinical evaluation in diabetic PNP detection [25]. In our study, the TCSS demonstrated a strong discriminative ability for neuropathy diagnosis (AUC 0.816), supporting its position as a practical and predictable screening tool in current medical practice. Additionally, Sudoscan sudomotor testing slightly improved the predictive performance not just in research, but in clinical models, too. This suggests that functional autonomic testing may provide complementary diagnostic information.

In other words, much more briefly, the results suggest the following:

1. Age and PNP demonstrated that each additional year of life was associated with a 6% increase in the odds of PNP.
2. The HbA1c and PNP progression association suggests that a poorer glycemic control contributed meaningfully to an increment in the PNP risk.
3. The TCSS score, a measure that reflects the clinical severity, was strongly associated with PNP.
4. Neurotrophic treatment was recommended in a significant proportion of patients, most likely reflecting its administration in more severe cases of PNP.
5. The association of BMI and WC with PNP suggests that they have potential clinical relevance that warrants further investigation.

The study limitations include the small sample and the unicentric design, while its strengths include its aim to find predictors for PNP prediction. Despite these limitations, the trends observed in the present study are consistent with established theories regarding metabolic contributions to neuropathy. The marginal significance of anthropometric predictors underscores the need for larger, longitudinal studies to further clarify their role.

As future directions, prospective longitudinal studies incorporating multiple variables that quantify advanced adiposity indices, inflammatory biomarkers, and neurophysiological measures are needed to clarify temporal relationships and refine predictive models. Also, integrative approaches combining metabolic, vascular, and inflammatory parameters may provide superior risk stratification and create targeted therapeutic strategies.

## 5. Conclusions

This study demonstrates that age and clinical assessment of PNP through TCSS are strongly associated with the presence of PNP in patients with type 2 DM, while glycemic control as measured by HbA1c, anthropometric parameters evaluated by BMI and WC, and the use of neurotrophic treatment show weaker or borderline association in patients with metabolic disease. So, anthropometric risk factors merit further investigation, particularly in the context of visceral adiposity and systemic inflammation.

The modest improvement in model performance with the inclusion of sudomotor assessment using Sudoscan, a functional testing tool, suggests that it may provide complementary information in the clinical evaluation of diabetic PNP.

However, taking into consideration the methodological constraints of the study design—specifically, the cross-sectional design and the impossibility of establishing a temporal relationship between the moment of variable evaluation and the development of PNP—the observed association should not be interpreted as evidence of causality, but rather as markers of a potential relationship that may aid to identify patients at increased clinical risk, improving clinical decision-making and mitigating complications associated with neuropathy.

So, until future longitudinal and prospective studies are conducted that incorporate metabolic, inflammatory, and neurophysiological parameters, which should establish the causal pathways and determine which factors contribute directly to the development and progression of PNP, the present results should primarily be considered as hypothesis-generating and supportive of risk stratification rather than causal inference in clinical practice.

## Figures and Tables

**Table 1:** Main characteristics of the studied group.

Parameter	
Age (years) (mean ± SD)	59.12 ± 1.24
Male gender (*n*, %)	63, 53.8%
Urban settlement (*n*, %)	95, 81.2%
T2DM (*n*, %)	98, 83.8%
DM time from onset (years) (mean ± SD)	9.85 ± 0.67
BMI (mean ± SD)	30.04 ± 0.50
Waist circumference (mean ± SD)	101.15 ± 1.37
HBA1c (mean ± SD)	8.38 ± 0.18
Total cholesterol (mg/dL) (mean ± SD)	191.82 ± 4.36
LDL-cholesterol (mg/dL) (mean ± SD)	115.74 ± 3.87
Triglycerides (mg/dL) (mean ± SD)	194.50 ± 30.85
Diabetic retinopathy (*n*, %)	34, 29.1%
Diabetic nephropathy (*n*, %)	11, 9.4%
Autonomic diabetic neuropathy (*n*, %)	45, 38.5%
PNP (*n*, %)	71, 60.7%
TCSS score (mean ± SD)	
History of stroke (*n*, %)	23, 19.7%
PNP treatment (%)	30, 25.6%
Chronic coronary syndrome (*n*, %)	67, 57.3%
History of amputation (*n*, %)	7, 6%
Peripheral artery disease (*n*, %)	21, 17.9%
Chronic venous disease (*n*, %)	37, 31.6%
History of trophic lesions or ulcerations (*n*, %)	21, 17.9%

SD—standard deviation; T2DM—type 2 diabetes mellitus; DM—diabetes mellitus; BMI—body mass index; HbA1c—glycated hemoglobin. LDL-cholesterol—low-density-lipoprotein cholesterol; PNP—peripheral neuropathy; TCSS—Toronto Clinical Severity Score.

**Table 2:** Comparison between patients with and without peripheral neuropathy.

Parameter	With PNP (*n* = 71)	Without PNP (*n* = 46)	*p*-Value
Age (years) (median [IQR])	65 (57–69)	59.50 (46–68)	*p* = 0.008
Male gender (%)	54.9%	52.2%	*p* = 0.770
Urban settlement (%)	85.9%	73.9%	*p* = 0.105
T2DM (%)	88.7%	76.1%	*p =* 0.070
DM time from onset (years) (median [IQR])	10 (6–15.50)	5.5 (2–14)	*p* = 0.019
BMI (kg/m^2^) (mean ± SD) Waist circumference (cm) (mean ± SD) HbA1c (%) (mean ± SD)	30.07 ± 0.65	30 ± 0.80	*p* = 0.954
	101.38 ± 1.69	101.78 ± 2.32	*p* = 0.832
	8.49 ± 0.25	8.21 ± 0.25	*p* = 0.449
Total cholesterol (mg/dL) (mean ± SD) LDL-cholesterol (mg/dL) (mean ± SD)	177.20 ± 5.28	188.94 ± 7.46	*p* = 0.190
	110.53 ± 4.43	123.78 ± 6.98	*p* = 0.113
Triglycerides (mg/dL) (median [IQR])	204.33 (102.50–207.50)	147.50 (89–193)	*p* = 0.708
Diabetic retinopathy	31%	26.1%	*p* = 0.569
Diabetic nephropathy	12.7%	4.3%	*p* = 0.197
Autonomic diabetic neuropathy	56.3%	10.9%	*p* < 0.001

PNP—peripheral neuropathy; IQR—interquartile range; T2DM—type 2 diabetes mellitus; DM—diabetes mellitus; BMI—body mass index; SD—standard deviation; HbA1c—glycated hemoglobin; LDL-cholesterol—low-density-lipoprotein cholesterol.

## Data Availability

Data supporting the reported results can be obtained on request from the authors, due to the fact that this is an ongoing doctoral project that still has results to be published.

## Abbreviations

The following abbreviations are used in this manuscript:

BMI	Body mass index
DM	Diabetes mellitus
EPV	Events Per Variable
MRI	Magnetic resonance imaging
PNP	Peripheral neuropathy
TCSS	Toronto Clinical Scoring System
WC	Waist circumference
